# The Effect of Eplerenone on Adenosine Formation in Humans *In Vivo*: A Double-Blinded Randomised Controlled Study

**DOI:** 10.1371/journal.pone.0111248

**Published:** 2014-10-30

**Authors:** T. N. A. van den Berg, Jaap Deinum, Albert Bilos, A. Rogier T. Donders, Gerard A. Rongen, Niels P. Riksen

**Affiliations:** 1 Department of Pharmacology-Toxicology, Radboud University Medical Center, Nijmegen, the Netherlands; 2 Department of Internal Medicine, Radboud University Medical Center, Nijmegen, the Netherlands; 3 Department for Health Evidence, Radboud University Medical Center, Nijmegen, the Netherlands; Kurume University School of Medicine, Japan

## Abstract

**Background:**

It has been suggested that mineralocorticoid receptor antagonists have direct cardioprotective properties, because these drugs reduce mortality in patients with heart failure. In murine models of myocardial infarction, mineralocorticoid receptor antagonists reduce infarct size. Using gene deletion and pharmacological approaches, it has been shown that extracellular formation of the endogenous nucleoside adenosine is crucial for this protective effect. We now aim to translate this finding to humans, by investigating the effects of the selective mineralocorticoid receptor antagonist eplerenone on the vasodilator effect of the adenosine uptake inhibitor dipyridamole, which is a well-validated surrogate marker for extracellular adenosine formation.

**Methods and Results:**

In a randomised, double-blinded, placebo-controlled, cross-over study we measured the forearm blood flow response to the intrabrachial administration of dipyridamole in 14 healthy male subjects before and after treatment with placebo or eplerenone (50 mg bid for 8 days). The forearm blood flow during administration of dipyridamole (10, 30 and 100 µg·min^−1^·dl^−1^) was 1.63 (0.60), 2.13 (1.51) and 2.71 (1.32) ml·dl^−1^·min^−1^ during placebo use, versus 2.00 (1.45), 2.68 (1.87) and 3.22 (1.94) ml·dl^−1^·min^−1^ during eplerenone treatment (median (interquartile range); *P* = 0.51). Concomitant administration of the adenosine receptor antagonist caffeine attenuated dipyridamole-induced vasodilation to a similar extent in both groups. The forearm blood flow response to forearm ischemia, as a stimulus for increased formation of adenosine, was similar during both conditions.

**Conclusion:**

In a dosage of 50 mg bid, eplerenone does not augment extracellular adenosine formation in healthy human subjects. Therefore, it is unlikely that an increased extracellular adenosine formation contributes to the cardioprotective effect of mineralocorticoid receptor antagonists.

**Trial Registration:**

ClinicalTrials.gov, NCT01837108

## Introduction

Despite state-of-the-art reperfusion strategies, mortality and morbidity in patients with an acute myocardial infarction remain significant. This is caused, at least in part, by ’lethal reperfusion injury’ [1]. Therefore, novel therapeutic options to further limit ischemia-reperfusion (IR) injury are urgently needed to improve outcome in these patients.

It has been suggested that the mineralocorticoid receptor (MR) antagonists spironolactone and eplerenone could potentially serve this goal, because these drugs reduce mortality in patients with heart failure [2]–[4]. Indeed, recent studies in murine models of myocardial infarction have shown that MR antagonists can directly limit infarct size [5]–[8], and have beneficial effects on left ventricular remodeling [9], [10].

The underlying mechanisms of the infarct size-limiting effect are not yet fully understood, but it has been suggested that the endogenous purine nucleoside adenosine is crucially involved. Adenosine is an endogenous purine nucleoside, which is formed by intra-, and extracellular degradation of adenosine monophosphate by the enzyme ecto-5’-nucleotidase (CD73). Degradation of adenosine only occurs in the intracellular compartment. As a consequence, facilitated diffusion of adenosine over the cellular membrane by the equilibrative nucleoside transporter (ENT) is normally directed inwards. Stimulation of membrane-bound adenosine receptors (A_1_, A_2A_, A_2B_, and A_3_) induces various effects, including vasodilation, inhibition of inflammation, and protection against IR-injury. Indeed, endogenous adenosine acts as a key mediator of the infarct size-limiting effect of several drugs, including statins and metformin [11], [12]. A recent series of experiments, using genetic and pharmacological approaches in mouse and rat models of myocardial infarction, convincingly demonstrated that the cardioprotective effects of the MR antagonists eplerenone and canrenoate were crucially dependent on extracellular adenosine formation by CD73 and adenosine receptor stimulation [6].

Based on these previous animal studies, we now hypothesize that MR antagonists increase the extracellular adenosine concentration by activating CD73, which has previously also been reported for statins [13], and we aim to test this hypothesis in humans *in vivo*. Measurement of circulating endogenous adenosine is extremely difficult [14], because the half life of adenosine in blood is very short, due to rapid uptake and degradation by circulating erythrocytes [15]. Therefore, we used the vasodilator effect of the ENT inhibitor dipyridamole as a read-out for endogenous adenosine formation by CD73, as previously described by our group [16], [17]. The results of this study will give novel insight into the pharmacology of MR antagonists, which can be used to optimize pharmacological cardioprotective strategies, for example avoid the use of adenosine receptor antagonists, such as caffeine, in patients treated with MR antagonists.

## Methods

The protocol for this trial and supporting CONSORT checklist are available as supporting information; see Checklist S1 and Protocol S1.

### Study population

After approval by the Institutional Review Board of our centre, we included 14 healthy male volunteers. They had no history of cardiovascular disease or asthma, did not smoke and did not use any medication. In all participants we performed a physical examination, electrocardiography, and laboratory investigation to exclude cardiovascular and pulmonary disease, hypertension (SBP >140 mmHg or DBP >90 mmHg), hypotension (SBP <100 mmHg or DBP <60 mmHg), diabetes mellitus (fasting venous glucose >6.9 mmol/l or random glucose of >11.0 mmol/l), renal dysfunction (MDRD <60 mL/min), liver enzyme abnormalities (alanine aminotransferase (ALAT) twice upper limit), and hyperkalemia (plasma potassium ≥4.8 mmol/l).

All volunteers provided written informed consent before enrollment. The study was conducted in accordance with Good Clinical Practices and the Declaration of Helsinki and was prospectively registered at ClinicalTrials.gov by number NCT01837108.

### Study design

We performed a single center, double-blinded, randomised, placebo-controlled cross-over study. Tablets of 50 mg of eplerenone were over-encapsulated and fully mimicking placebos were created by the Department of Clinical Pharmacy of the Radboud University Medical Centre. Randomization was performed by the Department of Clinical Pharmacy and the randomization code was kept at this department until after analyses and locking of the database. Study medication was taken bid during 8 days. We advised the participants to have a diet low in potassium. On the 7^th^ and 8^th^ day of treatment, we measured forearm blood flow (FBF) with the use of venous occlusion plethysmography. After a wash-out period of at least 4 weeks, the participants crossed-over to the alternative treatment arm. Blood pressure was measured at baseline, on day 3–5, on day 7 and 8.

### Venous occlusion plethysmography

All experiments were performed in a temperature-controlled room (24±0.5°C), in the morning after an overnight fast and at least 24 hours of caffeine and alcohol abstinence. On the days of the experiments, 75 minutes after supervised intake of the study drug, a 27-gauge needle (B. Braun Medical B.V.) was inserted into the brachial artery of the non-dominant arm for intra-arterial drug administration. Fifteen minutes later, baseline FBF was measured during the infusion of normal saline. During the experiment, the total volume infused into the brachial artery was kept constant at 100 µl·min^−1^·dl^−1^ of forearm volume. We measured FBF in both arms with venous occlusion plethysmography, using mercury-in-silastic-strain gauges. The hand circulation was occluded during the measurements, as described previously [18]. Drugs were administered for 5 minutes per dose. On day 7, we performed 3 experiments, which were all separated by a wash-out period of 30 minutes to prevent any cross-over effects.

1. We measured FBF during the administration of incremental dosages of dipyridamole (10, 30 and 100 µg·min^−1^·dl^−1^ of forearm volume) into the brachial artery (10).
2. Subsequently, we measured the vasodilator response to 2 minutes and 5 minutes of arterial occlusion (‘post-occlusive reactive hyperemia’ (PORH)). Forearm ischemia was induced by inflation of an upper arm cuff to 200 mmHg, as described previously [13]. PORH was used as a stimulus for increased endogenous extracellular adenosine formation.
3. Finally, on day 7, we measured FBF during simultaneous administration of dipyridamole (10, 30 and 100 µg·min^−1^·dl^−1^) and the adenosine receptor antagonist caffeine (90 µg·min^−1^·dl^−1^) into the brachial artery.

**Figure 1:**
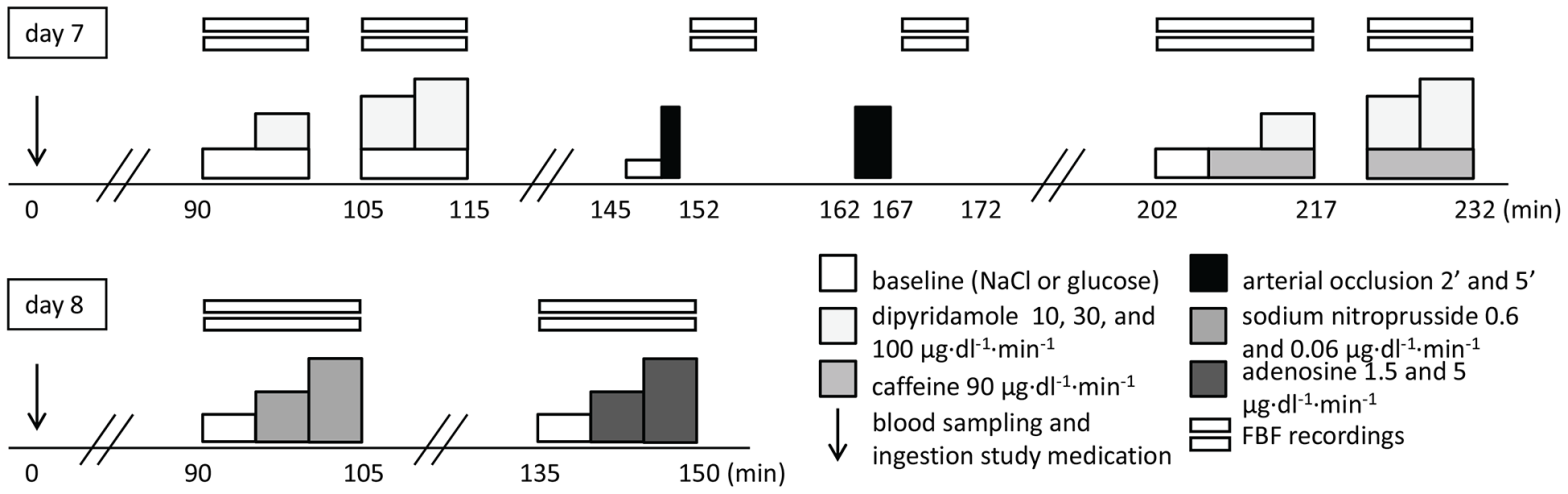
Schematic overview of the experimental protocol.

On day 8, we recorded the forearm vasodilator response to the administration of sodium nitroprusside (SNP) (0.6 and 0.06 µg·min^−1^·dl^−1^) and adenosine (1.5 and 5.0 µg·min^−1^·dl^−1^) to exclude non-specific effects of eplerenone on vasomotor function and adenosine sensitivity. Figure 1 illustrates the design of the study.

### Blood and urine sampling

Three to 5 days after the start of the study medication, the plasma potassium concentration was measured in venous blood. Volunteers were excluded and study medication was discontinued if the serum potassium was ≥5.1 mmol/l.

At day 6, the participants collected a 24-hours urine sample. Urinary sodium and creatinine were determined to ensure that salt intake was approximately the same during both treatment periods.

Before the experiment on day 7, blood was drawn for the determination of potassium, sodium, creatinine, plasma caffeine concentration (to check compliance with caffeine abstinence), aldosterone, renin, and plasma eplerenone concentration. On day 8 we measured the plasma caffeine concentration only. Subjects with a circulating caffeine concentration >1.0 mg/l were excluded from analyses.

### Analytic procedures

Plasma caffeine concentrations were determined using reversed-phase HPLC with ultraviolet detection set at 273 nm, according to Schreiber-Deturmeny and Bruguerolle [19]. After data monitoring and data lock, eplerenone concentrations were determined by LC-MSMS. Liquid chromatographic separation was performed at a temperature of 30°C with a mobile phase consisting of solvent A (0.1% (v/v) formic acid (HCOOH) in water) and solvent B (0.1% (v/v) HCOOH in acetonitrile). For the mass spectrometric analysis, heated electrospray ionization was operated at a spray voltage of +4.5 kV, a capillary temperature of 225°C and a vaporizer temperature of 382°C. Positive ion mode was used with selected reaction monitoring for the quantitative analysis of eplerenone, using the most abundant product ion for quantification.

### Outcomes

The primary outcome was the FBF response to the intrabrachial administration of incremental dosages of dipyridamole, after treatment with eplerenone, compared to placebo.

Secondary outcomes were the FBF response to the intrabrachial administration of incremental dosages of dipyridamole with concomitant administration of caffeine, and the FBF response to incremental periods of arterial occlusion.

### Statistical analysis

For sample size calculation, we assumed a 25% increase in the primary endpoint, a standard deviation of the logarithm of the FBF of 0.35, and a correlation between both experiments of 0.7. This assumption was based on the previous findings that MR antagonists reduced infarct size in preclinical studies with approximately 30% [6], and that rosuvastatin, which increases CD73 activity with approximately 50% approximately doubles dipyridamole-induced forearm vasodilation [13], [17]. The power of the study was set at 80% with a two-sided alpha of 0.05. As results, 12 evaluable subjects were needed. In order to have 12 evaluable subjects, we included 14 eligible participants.

FBF analyses were done offline before unblinding of the study. We averaged all individual FBF responses during the last 4 minutes of the baseline FBF (normal saline), the last 2 minutes of the FBF response to dipyridamole, sodium nitroprusside, and adenosine. For the PORH, we selected the highest FBF after reperfusion and also averaged FBF values for each consecutive minute. Results are expressed as the median absolute FBF (interquartile range) in ml·dl^−1^·min^−1^. In an additional analysis, to correct for any possible systemic effects on FBF during the experiments, we divided the FBF in the experimental arm by the FBF in the non-experimental arm. This analysis was performed with untransformed data.

A linear mixed model was used to compare differences between the treatments, with the log FBF during placebo and eplerenone treatment as the dependent variable with the following fixed factors: treatment (eplerenone versus placebo), dose, period, and the interaction between treatment and dose. To correct for repeated measurements, we used a heterogeneous compound symmetry structure for the residuals.

For the comparison of the plasma potassium values, blood pressure, and heart rate on a single moment as well as the measurements in the 24 hours urine samples and plasma sodium, serum aldosterone, and plasma renin values, we used a paired sample t-test, after testing for normality.

## Results

### Subjects

Screening was performed in the period 18-04-2013 until 22-10-2013 and the experiments were performed in the period from 08-05-2013 until 23-12-2013. We screened 20 subjects for eligibility. Two participants withdrew from participation and 4 participants were excluded, because of a DBP <60 mmHg (n = 3), and a SBP >140 Hg (n = 1). The baseline characteristics are depicted in Table 1.

**Table 1:** Baseline characteristics of the male participants.

Characteristic	Value
Age - years	21.9±3.0
BMI - kg/m^2^	23.6±1.9
Blood pressure - mmHg	
SBP	127±8
DBP	70±8
Heart rate – beats/min	59±9
Blood plasma	
Potassium - mmol/L	3.9±0.3
Creatinine - µmol/L	82.2±9.7
Non-fasting glucose - mmol/L	5.3±0.6
ALAT - U/L	30.0±5.3
Non-fasting cholesterol - mmol/L	3.8±0.6

**Figure 2:**
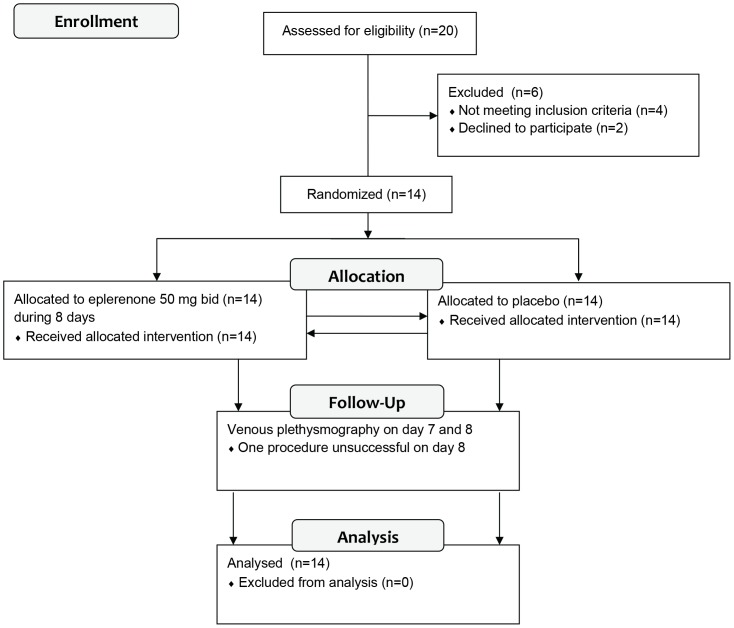
Consort 2010 flow diagram.

FBF responses to the different stimuli were obtained in all 14 subjects, with the exception of the FBF response to SNP and adenosine in 1 subject, because insertion of the arterial needle failed. All baseline plasma caffeine concentrations were below 0.6 mg/l, and therefore none of the subjects had to be excluded from analysis. Please see Figure 2 for the flow diagram of the various different phases of this cross-over trial.

Eplerenone treatment did not significantly affect blood pressure and serum potassium, but there was a significant decrease in the plasma sodium concentration (Table 2). Urinary sodium concentration did not significantly differ between placebo and eplerenone treatment. Furthermore, eplerenone treatment almost doubled the serum aldosterone and plasma renin concentrations (*p*<0.05), with an unchanged aldosterone-to-renin-ratio (Table 2; *p* = 0.30).

**Table 2:** Hemodynamic and laboratory values during the study.

	Placebo	Eplerenone	*p*-value
Blood plasma			
Potassium day 3–5 - mmol/L	3.9±0.26	3.9±0.28	0.78
Potassium day 7 - mmol/L	3.6±0.23	3.7±0.27	0.12
Sodium day 7 - mmol/L	140.0±1.4	139.1±1.4	<0.05
Creatinine day 7 -µmol/L	82.1±6.8	84.6±6.4	<0.05
Aldosterone day 7 - nmol/L	0.65±0.41	1.20±0.50	<0.05
Renin day 7- mE/L	27.7±18.1	44.8±26.7	<0.05
ARR	34.2±21.0	38.0±16.9	0.30
24 hr urine (day 6)			
Total amount - mL	1475.6±520.0	1635.4±677.4	0.34
Sodium - mmol/L	85.7±50.3	104.7±44.4	0.15
Creatinin - mmol/L	13.3±6.2	12.0±5.2	0.52
Blood pressure - mmHg			
SBP day 3–5	126±9	129±7	0.57
DBP day 3–5	65±7	67±11	0.54
SBP day 7	123±10	120±8	0.19
DBP day 7	62±10	62.6±8	0.58
SBP day 8	124±11	126±10	0.52
DBP day 8	65±8	63±6	0.34
Heart rate - beats per minute			
HR day 3–5	63±10	62±9	0.42
HR day 7	59±11	58±10	0.51
HR day 8	61±11	60±7	0.74

### Outcomes

**Figure 3:**
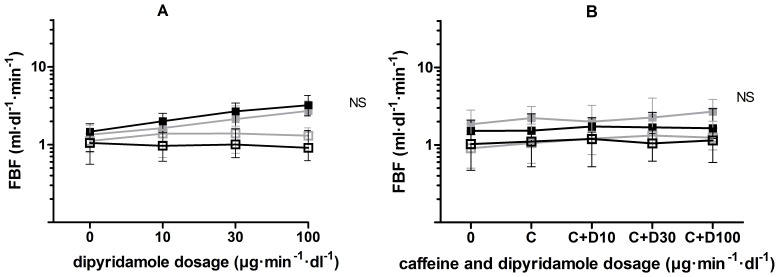
FBF response to dipyridamole, without and with caffeine. FBF response to **A** dipyridamole and **B** dipyridamole (D) in incremental dosages during concomitant administration of caffeine (C) in a constant dosage of 90 µg·dL^−1^·min^−1^, during placebo (grey) and eplerenone (black) treatment in the experimental (filled squares) and non-experimental (open squares) arm.

Baseline FBF before intra-arterial infusion of dipyridamole was 1.34 (0.82) ml·dl^−1^·min^−1^ during placebo and 1.47 (1.05) ml·dl^−1^·min^−1^ during eplerenone treatment. The incremental dosages of dipyridamole increased FBF in the experimental arm to 1.63 (0.60), 2.13 (1.51) and 2.71 (1.32) ml·dl^−1^·min^−1^, and 2.00 (1.45), 2.68 (1.87) and 3.22 (1.94) ml·dl^−1^·min^−1^ during placebo and eplerenone treatment, respectively. There was no significant increase in FBF response to dipyridamole during eplerenone treatment compared to the placebo experiment (Figure 3A; *p* = 0.51). Similarly, the FBF ratio did not differ between placebo and eplerenone treatment (*p* = 0.79). In none of the experiments changes in FBF in the non-experimental arm were observed.

Caffeine significantly blunted the dipyridamole-induced vasodilator response during placebo and eplerenone treatment (*p*<0.001), but there was no difference between both treatment periods (Figure 3B; *p* = 0.98).

**Figure 4:**
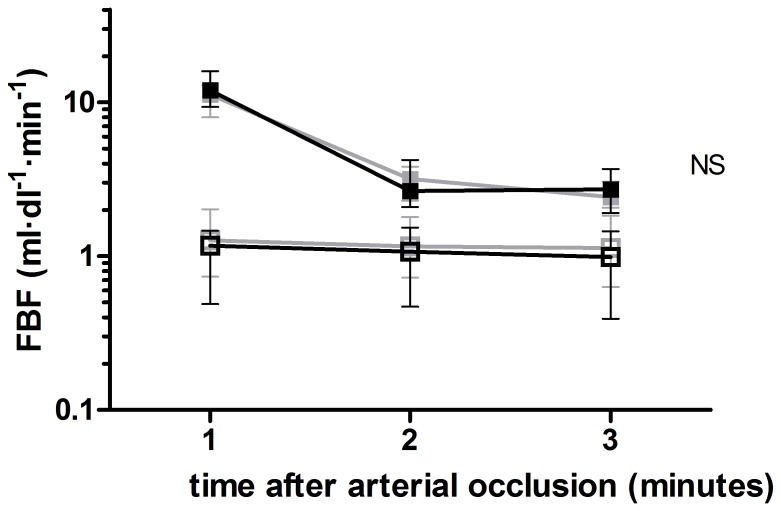
PORH after 2 minutes of arterial occlusion. PORH in the first 3 minutes after 2 minutes of arterial occlusion during placebo (grey) and eplerenone (black) treatment in the experimental (filled squares) and non-experimental (open squares) arm.

The peak (absolute) FBF’s after 2 and 5 minutes of arterial occlusion were 20.00 (9.73) and 27.6 (7.45) ml·dl^−1^·min^−1^ respectively during placebo, and 23.05 (12.35) and 27.75 (16.05) ml·dl^−1^·min^−1^ respectively during eplerenone use (*p* = 0.91). Figure 4 shows that the PORH after 2 minutes of arterial occlusion was not potentiated by eplerenone (*p* = 0.73). The averaged FBF after 5 minutes of arterial occlusion was 11.24 (4.94) in the 1^st^ minute, 3.18 (1.52) in the 2^nd^ minute, and 2.42 (0.99) ml·dl^−1^·min^−1^ in the 3^rd^ minute after arterial occlusion during placebo use. During eplerenone treatment, FBF was 11.97 (6.60), 2.65 (2.14), and 2.72 (1.78) ml·dl^−1^·min^−1^ in the first 3 minutes after 5 minutes of arterial occlusion. Eplerenone did not potentiate the PORH after 5 minutes of arterial occlusion (*p* = 0.58).

**Figure 5:**
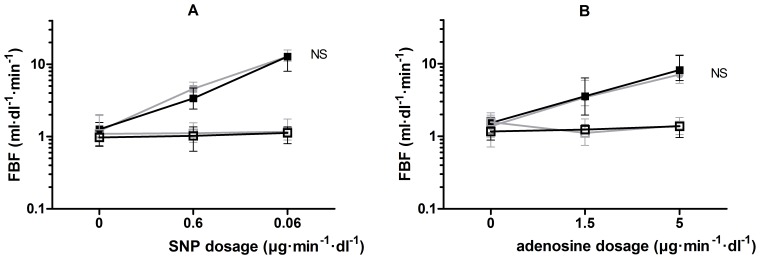
FBF response to sodium nitroprusside (SNP) and adenosine. The FBF response to **A** sodium nitroprusside (SNP) and **B** adenosine, during placebo (grey) and eplerenone (black) treatment in the experimental arm (filled squares) and non-experimental arm (open squares).

The vasodilator response to SNP and adenosine did not differ between placebo and eplerenone treatment (Figure 5).

The plasma eplerenone concentration on t = 0 was 0.17±0.13 µg/ml (mean ± SD). During placebo, the eplerenone concentration was 0.0 µg/ml in all cases.

### Safety

There were no serious adverse events. In none of the subjects, serum potassium exceeded 4.6 mmol/l. Thirteen adverse events occurred in 9 different participants. During eplerenone treatment 1 subject experienced a short period of abdominal pain on day 4 of the treatment and another subject had a mild headache on day 7. During placebo use 2 subjects had a short period of headache, 1 subject experienced light headedness during sports activities, 1 subject had symptoms of hay fever and another subject developed a skin rash. Due to dislocation of the arterial needle, a very small amount of the infused volume was administered extra-arterially in 3 subjects. Another subject developed 3 small, pulsating, yellowish hives just medial from the arterial needle during dipyridamole infusion, which resolved spontaneously. One subject experienced pain in his left shoulder during the experiment, which resolved after we repositioned the participant. Finally, 1 subject developed a forearm hematoma after several unsuccessful attempts to cannulate the brachial artery on day 8.

## Discussion

This study indicates that the selective MR antagonist eplerenone does not have an effect on the extracellular adenosine formation in humans *in vivo*, excluding this mechanism as an explanation for the beneficial cardiovascular effects of MR antagonism observed in patients with heart failure.

The Randomized Aldactone Evaluation Study (RALES), Eplerenone Post–acute myocardial infarction Heart failure Efficacy and Survival Study (EPHESUS), and Eplerenone in Mild Patients Hospitalization And Survival Study in Heart Failure (EMPHASIS-HF) showed that treatment with MR antagonists reduces the mortality and the number of hospitalizations in patients with mild to severe systolic heart failure [2]–[4]. In animal models of myocardial infarction, MR antagonists limit infarct size when administered either prior to ischemia or just before the onset of reperfusion, and protect against cardiac remodeling, as reviewed by van den Berg *et al*. [20].

The underlying mechanism of the beneficial effects of MR antagonists in patients is not yet understood. However, results from animal studies suggest that extracellular adenosine formation is crucial for the protective effect of MR antagonists against myocardial IR injury. In an elegant series of experiments in mice and rabbits, Schmidt et al. showed that the MR antagonists canrenoate and eplerenone reduce infarct size in a dose-dependent manner. Moreover, by using pharmacological approaches and models of targeted gene deletion, the investigators convincingly demonstrated an important role for endogenous adenosine in the cardioprotective effect. The infarct size-limiting effect of canrenoate was completely abolished in CD73 knock-out mice and in adenosine A_2b_ receptor knock-out mice. Similar results were obtained in isolated rat hearts. In rats, administration of eplerenone at 10 µM at the moment of reperfusion resulted in a reduction of the infarct size from 40 to 10%, which was completely blocked by co-administration of the adenosine receptor blocker 8p-sulfophenyladenosine [6].

Based on this series of experiments, we hypothesized that the selective MR antagonist eplerenone increases extracellular formation of adenosine, by activation of the enzyme CD73. Interestingly, increased endogenous adenosine receptor stimulation has also been implicated in the cardioprotective effect of other drugs, including statins, methotrexate and metformin [12], [21], [22].

It is difficult to investigate the potential effects of drugs on endogenous adenosine because the half life of adenosine in blood is extremely short due to rapid uptake and degradation of adenosine by erythrocytes and endothelial cells [16]. In a normal physiological situation, the transmembranous adenosine concentration gradient drives extracellular adenosine into the cytosol [23]. The ENT-inhibitor dipyridamole inhibits this facilitated diffusion and thereby increases the extracellular adenosine concentration, which can subsequently activate membrane-bound adenosine receptors at the site of adenosine formation [16], [24]. These observations justify the use of dipyridamole-induced vasodilation as a read-out of extracellular adenosine formation. We have previously shown, using a similar experimental design as the present study, that rosuvastatin augments dipyridamole-induced forearm vasodilation and post-occlusive reactive hyperemia and limits forearm ischemia-reperfusion injury via adenosine receptor stimulation [13], [17]. Moreover, rosuvastatin increased the activity of CD73 on circulating human mononuclear cells [13].

In the present study, we could not confirm a relation between treatment with eplerenone and the extracellular adenosine system in healthy humans *in vivo*. We showed that a one-week treatment with eplerenone 50 mg bid does not potentiate dipyridamole-induced vasodilation and does not affect PORH after 2 and 5 minutes of arterial occlusion. These findings are in sharp contrast to the previous observations in animal models of myocardial infarction [6].

How can this discrepancy be explained? First, it has been recognized that many promising findings in animal study cannot be confirmed in human studies [25]. In general, this translational failure can be explained by methodological shortcomings in animal studies, e.g. the lack of a formal sample size calculation, selection bias due to the lack of randomization, unblinded researchers, and inadequate statistical analyses [25]. The scientific basis for our hypothesis is based on only one preclinical study by Schmidt et al [6]. This study, however, described a series of experiments in which a pivotal role for endogenous adenosine in the infarct size-limiting effect of the MR antagonists canrenoate and eplerenone was consistently observed in different animal species and by using various methodological approaches, including pharmacological inhibitor studies and gene deletion models. Translational failure could also result from fundamental differences between the animal models and the human situation. With regard to adenosine metabolism, it is known for fundamental differences exists between rats and humans (e.g. the rat ENT1 transporter is less sensitive to inhibition with dipyridamole than the human ENT1) [26]. However, other drugs that modulate adenosine levels seem equally effective in humans and in animal models [17], [21], [22], [27]. Thirdly, a major difference in drug concentration between the animal studies and the human situation is often present. In our study, the circulating eplerenone concentration immediately before the intake of the last medication dose (trough concentration) averaged 0.17 µg/ml (0.41 µM). Given a bioavailability of 70%, and an apparent volume of distribution of 43–90 liters, the eplerenone concentration at the moment of the experiment is approximately 1.7 µM. In a rat Langendorff model, in which the heart is perfused with a buffer solution [6], a cardioprotective effect of eplerenone was observed with 10 µM, but not with 1 µM. Given a plasma protein binding of 50%, the effective concentration in the rat study is approximately 10-times higher than the concentration in our study. We used 50 mg of eplerenone bid, which is higher than the dosages administered in the clinical trials in patients with heart failure in which eplerenone showed beneficial effects [3], [4]. Therefore, we cannot exclude that eplerenone does affect adenosine formation at higher concentrations, which are not relevant however for the clinical situation. In addition, in our study, eplerenone was administered during one week, in contrast to the single dose administration in the animal studies. As expected, the serum aldosterone and plasma renine levels had almost doubled after 6 days of treatment with eplerenone. Although this increase in aldosterone does probably not affect our results, because eplerenone blocks the MR, MR-independent effects of aldosterone have been described previously (20). In theory, any non-MR-mediated effects of aldosterone on adenosine metabolism might have influenced our result. Finally, it is important to realize that our experiments were performed in healthy subjects, and not in patients with cardiovascular disease or heart failure. This was done because we aimed to demonstrate a general pharmacological mechanism of eplerenone and also because the preclinical studies that were fundamental to our hypothesis were performed in healthy animals. We cannot exclude, however, that the effects of MR antagonists are different in patients with cardiovascular disease, such as heart failure.

## Supporting Information

Checklist S1 **CONSORT Checklist.**
(DOCX)

Protocol S1 **Trial study protocol: final version of the study protocol approved by the Institutional Review Board.**
(PDF)
