# Supplementary material for: The Effect of Eplerenone on Adenosine Formation in Humans In Vivo: A Double-Blinded Randomised Controlled Study
Source: PLoS One. 2014 Oct 30;9(10):e111248. doi: 10.1371/journal.pone.0111248 (PMC4214740; doi:10.1371/journal.pone.0111248)
Supplement: Protocol S1 — Trial study protocol: final version of the study protocol approved by the Institutional Review Board. (PDF) [file pone.0111248.s002.pdf]

C1. NL43234_091_13

Effects of the selective mineralocorticoid receptor antagonist eplerenone on extracellular adenosine formation in humans in vivo

January 2013

Versie 2~~1~~, 26~~3~~ ~~januari~~ februari 2013

C1. NL43234_091_13

Protocol title: effects of the mineralocorticoid receptor (MR) antagonists on extracellular adenosine formation in humans in vivo

**Protocol ID**	NL43234_091_13
**Short title**	eplerenone and adenosine formation
**EudraCT number**	2013-000189-12
**Version**	2~~1~~
**Date**	2013 ~~January 23th~~February 26th
**Principal investigator(s) (in Dutch: hoofdonderzoeker/ uitvoerder)**	Theodora N.A. van den Berg, MSc<br>Dr. Niels P. Riksen<br>Prof. dr. Gerard Rongen<br><br>Dept. Of Pharmacology-Toxicology 149<br>Radboud University Nijmegen Medical Centre
**Sponsor (in Dutch: verrichter/opdrachtgever)**	Radboud University Nijmegen Medical Centre
**Subsidising party**	
**Independent expert (s)**	Drs. A. Rennings
**Laboratory sites**	Dept. of Pharmacology-Toxicology 149<br>Radboud University Nijmegen Medical Centre
**Pharmacy**	Dept. of Clinical Pharmacy<br>Radboud University Nijmegen Medical Centre

Met opmaak: Superscript

Protocol signature sheet

Name	Signature	Date
**Head of Department:**<br><br>Prof. dr. Frans Russel<br><br>Head of department of Pharmacology-Toxicology<br>Radboud University Nijmegen Medical Centre		
**Principal Investigators:**<br><br>Dr. Niels Riksen<br><br>Prof. dr. Gerard Rongen<br><br>Daniëlle van den Berg, MSc		

Table of contents

1. Introduction and rationale
2. Objectives
3. Study design
4. Study population
4.1 Population (base)
4.2 Inclusion criteria
4.3 Exclusion criteria
4.4 Sample size calculation
5. Treatment of subjects
5.1 Investigational product/treatment
5.2 Use of co-intervention (if applicable)
5.3 Escape medication (if applicable)
6. Investigational product
6.1 Name and description of investigational product(s)
6.2 Summary of findings from non-clinical studies
6.3 Summary of findings from clinical studies
6.4 Summary of known and potential risks and benefits
6.5 Description and justification of route of administration and dosage
6.6 Dosages, dosage modifications and method of administration
6.7 Preparation and labelling of Investigational Medicinal Product
6.8 Drug accountability
7. Non-investigational product
7.1 Name and description of non-investigational product(s)
7.2 Summary of findings from non-clinical studies
7.3 Summary of findings from clinical studies
7.4 Summary of known and potential risks and benefits
7.5 Description and justification of route of administration and dosage
7.6 Dosages, dosage modifications and method of administration
7.7 Preparation and labelling of Non Investigational Medicinal Product
7.8 Drug accountability
8. Methods
8.1 Study parameters/endpoints
8.1.1 Main study parameter/endpoint
8.1.2 Secondary study parameters/endpoints (if applicable)
8.1.3 Other study parameters (if applicable)
8.2 Randomisation, blinding and treatment allocation
8.3 Study procedures
8.4 Withdrawal of individual subjects
8.4.1 Specific criteria for withdrawal (if applicable)
8.5 Replacement of individual subjects after withdrawal

8.6 Follow-up of subjects withdrawn from treatment
8.7 Premature termination of the study
9. Safety reporting
9.1 Section 10 WMO event
9.2 AEs, SAEs and SUSARs
9.2.1 Adverse events (AEs)
9.2.2 Serious adverse events (SAEs)
9.2.3 Suspected unexpected serious adverse reactions (SUSARs)
9.3 Annual safety report
9.4 Follow-up of adverse events
9.5 [Data Safety Monitoring Board (DSMB) / Safety Committee]
10. Statistical analysis
10.1 Primary study parameter(s)
10.2 Secondary study parameter(s)
10.3 Other study parameters
10.4 Interim analysis (if applicable)
11. Ethical considerations
11.1 Regulation statement
11.2 Recruitment and consent
11.3 Objection by minors or incapacitated subjects (if applicable)
11.4 Benefits and risks assessment, group relatedness
11.5 Compensation for injury
11.6 Incentives (if applicable)
12. Adminstrative aspects, monitoring and publication
12.1 Handling and storage of data and documents
12.2 Monitoring and Quality Assurance
12.3 Amendments
12.4 Annual progress report
12.5 End of study report
12.6 Public disclosure and publication policy
13. Structured risk analysis
13.1 Potential issues of concern
13.2 Synthesis
14. References

List of abbreviations and relevant definitions

**ABR**	**ABR form, General Assessment and Registration form, is the application form t that is required for submission to the accredited Ethics Committee (in Dutch: ABR = Algemene Beoordeling en Registratie)**
**AE**	**Adverse Effect**
**AR**	**Adverse Reaction**
**bid**	**bis die (Latin): twice daily**
**CRF**	**Case Report Form**
**DBP**	**Diastolic Blood Pressure**
**DSMB**	**Drug Safety Monitoring Board**
**GCP**	**Good Clinical Practice**
**ENT**	**Equilibrative Nucleoside Transporter**
**FBF**	**Forearm BloodFlow**
**IR**	**Ischemia-Reperfusion**
**MI**	**Myocardial Infarcation**
**MR**	**Mineralocorticoid Receptor**
**NSAID**	**Non-Steroidal Anti-Inflammatory Drugs**
**PORH**	**Post-Occlusive Reactive Hyperemia**
**(S)AE**	**(Serious) Adverse Event**
**SBP**	**Systolic Blood Pressure**
**SNP**	**Sodium-Nitroprusside**
**TSH**	**Thyroid-Stimulating Hormone**

Summary

Rationale: Various studies have reported cardioprotective effects of mineralocorticoid receptor (MR) antagonists in the setting of an acute myocardial infarction. In a recent animal study, the protective effect of MR antagonists on infarct size was completely abolished in CD73 knock-out and adenosine A_{2b} receptor knock-out mice, and by co-administration of adenosine receptor antagonists in rats. These findings suggest that extracellular formation of adenosine is crucial for this protective effect and that MR antagonists stimulate extracellular adenosine formation by the enzyme CD73.

Objective: To investigate whether eplerenone promotes adenosine receptor stimulation by activating CD73

Study design: Single center, randomized, double-blinded, placebo-controlled, cross over study

Study population: Twelve + 2 healthy male volunteers, aged 18-40 years

Intervention (if applicable): All subjects will be randomized to treatment with either eplerenone (50 mg twice daily) or a placebo. Duration of the treatment will be 8 days for both eplerenone, and placebo. After a 4-week-period the subjects will be crossed over to the other treatment group.

Main study parameters: A venous cannula will be inserted into the antecubital vein of the dominant arm for blood drawing. The brachial artery of the non-dominant arm will be cannulated for local drug administration. We will measure forearm blood flow (FBF) with venous occlusion plethysmography. Subsequently, the FBF response will be measured to 1) the administration of incremental dosages of dipyridamole; 2) increasing periods of arterial occlusion; 3) concomitant administration of dipyridamole and caffeine and 4) two different concentrations of both adenosine and sodium-nitroprusside.

Primary analysis/endpoint: FBF, pooled over the various dosages of dipyridamole, will be compared between eplerenone and placebo.

Nature and extent of the burden and risks associated with participation, benefit and group relatedness: A physical examination, electrocardiography and blood sampling will be performed in all participants. All subjects will be treated with eplerenone (50 mg twice daily) and placebo. Potential side effects of eplerenone include nausea, diarrhoea, constipation, hypotension, myalgia, and hyperkalemia. The chance for these side-effects to occur is limited, because the study is restricted to young healthy males with a normal renal function, and the duration of treatment will be only 8 days. We will

reduce the risk of hyperkalemia by excluding volunteers with an estimated MDRD <60 mL/min/1.73 m^2 and a serum potassium ≥4.8 mmol/L. We will monitor serum potassium in the first week after start of study medication. Blood will be drawn 6 times during the study and 1 time before randomization. Volunteers will visit our clinic 9 times in total. On the four experimental days, a 27 gauge needle (outer diameter 0.4 mm) will be inserted into the brachial artery for local drug administration. This can cause a haematoma. The administration of dipyridamole, caffeine, adenosine and sodium-nitroprusside into the brachial artery has been performed many times previously by our study group, and is considered to be safe. There is no direct benefit for the participants from this study.

1. Introduction and rationale

Despite state-of-the-art reperfusion strategies, mortality and morbidity in patients with an acute myocardial infarction remain significant. This is caused, at least in part, by 'lethal reperfusion injury'. Therefore, novel therapeutic options to further limit ischemia-reperfusion (IR) injury are urgently needed to improve outcome in these patients.

It has been suggested that the mineralocorticoid receptor (MR) antagonists spironolactone and eplerenone could potentially serve this goal, because these drugs reduce mortality in patients with heart failure. Indeed, recent studies in murine models of myocardial infarction have shown that MR antagonists can directly limit infarct size. In more detail, in vitro studies and studies in animal models of myocardial infarction have reported that spironolactone, eplerenone, and canrenoate reduce myocardial infarction size (1-4) and protect against left ventricular remodeling (5, 6).

The underlying mechanisms of the infarct-size limiting effect are not yet fully understood, but it has been suggested that the endogenous purine nucleoside adenosine is crucially involved. In a recent animal study, canrenoate caused a dose-dependent reduction in infarct size. This protective effect of canrenoate was completely abolished in CD73 knock-out and adenosine A_{2b} receptor knock-out mice. In rats, eplerenone significantly reduced infarct size, and this beneficial effect was abolished by co-administration of adenosine receptor antagonists. (2) These findings suggest that extracellular formation of adenosine is crucial for the protective effect of MR antagonists on IR injury.

Adenosine is an endogenous purine nucleoside, which is formed by intra-, and extracellular degradation of adenosine monophosphate by the enzyme ecto-5'-nucleotidase, which is also named CD73. Degradation of adenosine occurs in the intracellular compartment. As a consequence, facilitated diffusion of adenosine over the cellular membrane by the equilibrative nucleoside transporter (ENT) is normally directed inwards. Stimulation of membrane-bound adenosine receptors induces various effects, including vasodilation, inhibition of inflammation, and protection against IR-injury. Indeed, endogenous adenosine acts as a key mediator of the infarct size-limiting effect of several drugs.

Measurement of adenosine is extremely difficult (7), because the half life of adenosine in blood is approximately one second, due to rapid uptake and degradation. (8) Therefore,

we have used previously validated and used alternative methods to indirectly study the endogenous adenosine concentration. Dipyridamole increases the extracellular endogenous adenosine concentration by inhibition of the ENT transporter (9) and induces local vasodilation. (10) Therefore, the vasodilator effect of dipyridamole accurately reflects extracellular adenosine formation by the CD73 enzyme. (11)

Our group has recently used this method to demonstrate that statins also augment CD73 activity. Brief arterial occlusion of the forearm is an endogenous stimulus to increase adenosine formation, which subsequently enhances blood flow, resulting in so-called 'post-occlusive reactive hyperemia' (PORH). This effect is also potentiated by dipyridamole and prevented by co-administration of caffeine. (12)

In this research proposal, we aim to investigate in humans in vivo whether eplerenone promotes adenosine receptor stimulation by activating CD73. The results of these studies will provide a possible explanation for the positive effects of MR antagonists on IR-injury.

To test our hypotheses, we will use the vasodilator response in the forearm vascular bed to various stimuli as a well-validated surrogate of adenosine receptor stimulation, as will be explained in more detail in the next section.

2. Objectives

Primary objective:

To study whether the MR antagonist eplerenone activates CD73 and hereby increases extracellular formation of adenosine in humans in vivo, by using the forearm vasodilator response to the intrabrachial administration of the ENT-inhibitor dipyridamole.

Secondary objective:

To study whether eplerenone increases adenosine formation, by measuring forearm blood flow to incremental periods of arterial occlusion, as an endogenous stimulus for adenosine upregulation.

3. Study design

In this randomized placebo-controlled double-blinded cross-over study, we will test the following hypotheses:

1. Eplerenone augments the vasodilator response to dipyridamole by activation of CD73
2. The adenosine receptor antagonist caffeine attenuates this augmented vasodilator response to dipyridamole.
3. Eplerenone augments postocclusive reactive hyperemia (PORH)

We chose to use eplerenone over spironolactone, because eplerenone is a selective MR antagonist without binding to the androgen and progesterone receptor, and thus without endocrine side effects. (See also paragraph 6.4 Summary of known and potential risks and benefits)

The design of the study is depicted in figure 1. In brief, we will ask 14 healthy male volunteers (age 18-40 years) to participate. After signing for informed consent, history taking, a physical examination, blood drawing (5 mL for creatinine, potassium, ALAT, total cholesterole and glucose) and electrocardiography will be performed. We would like to refer to paragraph 4.2 and 4.3 for the in-, and exclusion criteria.

After inclusion, the volunteers will be randomly allocated to start receiving either an 8-day treatment with eplerenone or fully mimicking placebo, in a double-blinded design. The

justification of the dose and timing of administration is given in chapter 6. In the first week of treatment (on day 3, 4 or 5 –depending on the weekend-) we will monitor serum potassium by drawing 3 mL of blood, in order to guarantee the safety of treatment with eplerenone. We accept a serum potassium to 5.0 mmol/L (in contrast to 4.7 for inclusion in the study) during the study. In case of a serum potassium of ≥ 5.1 mmol/L, the subject will be excluded from the study and the treatment will be stopped. Two days later, serum potassium will be evaluated again.

On the 7th day of treatment, the vasodilator response to intra-arterial administration of 3 increasing dosages of dipyridamole (10, 30 and 100 ug/min/dL per forearm) will be evaluated, in the absence and presence of intra-arterial infusion of caffeine (90 ug/min per 100 mL of forearm volume). In addition, PORH to 2 incremental periods of forearm ischemia (2 and 5 minutes) will be measured. Forearm ischemia will be induced by inflation of an upper arm cuff to 200 mmHg. During a separate visit (day 8), the vasodilator responses to intra-arterially infused sodium nitroprusside (60 and 600 ng/min/dL) and adenosine (1.5 and 5.0 ug/min/dL) will be determined, to exclude non-specific effects of eplerenone on vascular reactivity and adenosine sensitivity.

Treatment duration of 8 days means administration of eplerenone or placebo twice daily during 7 days and once daily on day 8.

After this first experiment, a wash out period of 4 weeks will avoid any carry-over effect of eplerenone. After these 4 weeks, the participant will be crossed over to receive the alternative treatment arm during 8 days. Again, we will monitor serum on day 3, 4 or 5, and on day 7 and 8 exactly the same experiments will be performed.

The subjects will be studied in supine position after an overnight fast and at least 24 h of caffeine and alcohol abstinence (since caffeine is an adenosine receptor antagonist and alcohol can also affect the adenosine metabolism). Experiments are performed in a temperature-controlled room (24 °C) in the morning after an overnight fast. At the start of the experiment, we will insert a venous cannula into the antecubital vein of the dominant arm for blood drawing. Blood will be drawn before start of each experiment: 20.5 mL on

day 7 for the determination of the serum potassium, sodium, creatinine, plasma caffeine (to check compliance with caffeine abstinence), aldosterone, renin, and eplerenone concentration; and 3 mL on day 8 for the determination of plasma caffeine only. Subjects with a circulating caffeine concentration > 1.0 mg/l will be excluded from analyses. Twenty-four hours urine samples will be collected and sodium and creatinine will be determined, to ensure that salt intake is approximately the same during both treatment days.

Study medication will be taken under supervision of the researcher. One hour after ingestion of the drug, a 27-gauge needle (B. Braun Medical B.V.) will be inserted into the brachial artery of the non-dominant arm for intra-arterial drug administration. The outer diameter of this needle is 0.4 mm. In both arms, forearm blood flow (FBF) will be measured simultaneously with venous occlusion plethysmography, using mercury-in-silastic-strain gauges and occluded hand circulation as described previously. (12) Thirty minutes after cannulation of the brachial artery, normal saline is infused with concomitant measurement of baseline FBF for 5 min.

So, approximately 1.5 hours after ingestion of the study medication, the experiment will be started and FBF will be measured around the T_{max} of eplerenone (approximately 2 hours) Three experiments will be performed, which are all separated by 30 minutes of wash-out.

1. FBF will be measured during the administration of increasing dosages of dipyridamole into the brachial artery. These dosages are similar to a previous study of our group. (10) We hypothesize that in patients treated with eplerenone, the dipyridamole-induced vasodilation is potentiated due to upregulation of CD73.
2. Subsequently, we will measure the vasodilator response to 2 incremental periods of arterial occlusion (‘post-occlusive reactive hyperemia’). Forearm ischemia will be induced by inflation of an upper arm cuff to 200 mmHg, as described previously. (11) We have previously demonstrated that the PORH after 2 and 5 minutes of ischemia is potentiated by statins (by increasing the extracellular formation of adenosine) (11) and by dipyridamole (by inhibition of the ENT transporter). (12)

3. We will finish the experiment on day 7 with measurement of FBF during the concomitant administration of the three increasing dosages of dipyridamole and of caffeine (90 ug/min per 100 mL of forearm volume) into the brachial artery.
4. Finally, on day 8, we will record the forearm vasodilator response to the administration of adenosine (1.5 and 5.0 ug/min/dL) and SNP (60 and 600 ng/min/dL) to exclude non-specific effects of eplerenone on vascular function and adenosine sensitivity.

Furthermore, we will measure blood pressure at baseline and during the experiments, on day 7 and 8, in all volunteers.

4. Study population

4.1. Population (base)

We will recruit 14 healthy male volunteers, aged 18-40 years. We will only include male patients to exclude any potential effect of circulating hormones on our outcome parameters, and to exclude an effect of mineralocorticoid receptor antagonists on foetal development in case of pregnancy.

The subjects will be recruited by advertisements placed throughout the university campus, the university sports centre, student flats, and placed on the university Intranet site.

Given our experience with previous studies with healthy male subjects, we will expect no problems recruiting 14 subjects for this study.

4.2. Inclusion criteria

In order to be eligible to participate in this study, a subject must meet all of the following criteria:

- Male sex
- Age 18-40 years
- Healthy
- Written informed consent

4.3. Exclusion criteria

A potential subject who meets any of the following criteria, will be excluded from participation in this study:

- Smoking
- Hypertension (Blood pressure >140 mmHg and/or >90 mmHg – SBP/DBP-)
- Hypotension (Blood pressure <100 mmHg and/or <60 mmHg –SBP/DBP-)
- Diabetes Mellitus (fasting glucose > 6.9 mmol/L or random > 11.0 mmol/L in venous plasma)

- History of any cardiovascular disease
- Angina pectoris
- History of chronic obstructive pulmonary disease (COPD) or asthma
- Alcohol and/or drug abuse
- Concomitant use of medication
- Renal dysfunction (MDRD < 60 ml/min/1.73 m^2)
- Liver enzyme abnormalities (ALAT > twice upper limit of normality)
- Serum potassium ≥ 4.8 mmol/L
- Fasting total cholesterol > 6.0 mmol/L
- Second/third degree AV-block on electrocardiography

During the study, we will monitor serum potassium again, on the 3rd, 4th or 5th day (depending on the weekend) of taking study medication. We will exclude subjects with a:

- Serum potassium ≥ 5.1 mmol/L

4.4. Sample size calculation (performed by Prof. Borm, dept. of Health Evidence)

Based on earlier, similar studies with a parallel groups design, we expect that the standard deviation of the logarithm of the FBF will be approx. 0.35 and that the correlations between the measurements within each period of the cross-over will be approx. 0.7. (12, 13) Based on these assumptions and a difference of 25% between the groups, the analysis described in the statistical section of the protocol, carried out on two parallel groups of 12 subjects, is expected to have approximately 80% power (two-sided testing at 0.05; the design factor for a pooled analysis on k follow-up measurements adjusted for baseline is $\frac{1}{k} + \frac{k-1}{k}\rho - \rho^2$). The proposed study is a cross-over trial, so the power of a 12-subject-study may be higher, as the subjects serve as their own control. However, this is to some extent also the case in a parallel study, because the baseline is included as a covariate in the analysis. Hence, the power of a cross over study with 12 evaluable subjects is expected to be only marginally higher than 80%. In order to have 12 evaluable subjects, we will enroll 14 subjects. (see also paragraph 8.5)

5. Treatment of subjects

5.1. Investigational product/treatment

All subjects will receive eplerenone 50 mg bid or matching placebo in a randomized double-blinded cross-over design.

5.2. Use of co-intervention (if applicable)

No co-medication is allowed. The experiments will be performed in the morning after an overnight fast and at least 24 hours of alcohol and caffeine abstinence, because caffeine is an effective adenosine receptor antagonist.

5.3. Escape medication (if applicable)

Not applicable

6. Investigational product

6.1 Name and description of investigational product(s)

Subjects will receive either eplerenone or a placebo. Eplerenone is a selective mineralocorticoid receptor antagonist. In the distale tubulus of the nephron it inhibits sodium exchange for potassium. Metabolism of eplerenone is mediated by CYP3A4. Maximal plasma concentration occurs 2 hours after ingestion and half life is 3-5 hours. Eplerenone is currently indicated in heart failure (after myocardial infarction and chronic heart failure). See also the attached SPC.
In the USA and Japan, eplerenone has also been aproved for primary hypertension. In addition, current guidelines of the Endocrine Society recommend spironolactone, as the primary agent, with eplerenon as an alternative treatment for patients with primary hyperaldosteronism. (14)

6.2 Summary of findings from non-clinical studies

We would like to refer to the attached SPC.
The role of MR antagonists in IR injury in non-clinical studies has been investigated in rats, mice, and pigs. In the past decade, several studies show that MR antagonists can reduce myocardial infarct size. Only one study explored the role of adenosine in the cardioprotective effects. (2) In this study, canrenoate (the active metabolite of spironolactone) caused a dose-dependent reduction in infarct size in mice. This protective effect of canrenoate was completely abolished in CD73 and adenosine A_{2b} receptor knock-out mice. Eplerenone showed a similar protective effect on infarct size in rats, but after (co-)administration of adenosine receptor antagonists, this cardioprotective effect was abolished. (2) These findings suggest that extracellular formation of adenosine is crucial for the protective effect of MR antagonists in IR.

In other animal studies, beneficial effects of MR antagonists on infarct size, cardiac remodeling (collagen deposition and interstitial fibrosis), myocyte apoptosis, left ventricular function and cardiac dilation are shown. (see chapter 1. Introduction and rationale)

6.3 Summary of findings from clinical studies

The large EPHESUS study included over 6600 patients and investigated the effect of eplerenone versus placebo in addition to optimal standard therapy on morbidity and mortality among patients with acute myocardial infarction, complicated by left ventricular dysfunction and heart failure. (15) The addition of eplerenone at a maximal dose of 50 mg once daily resulted in additional reductions in overall and cardiovascular mortality, and the rate of death from cardiovascular causes or hospitalization. For studies with eplerenone in healthy volunteers, please also see paragraph 6.5.

6.4 Summary of known and potential risks and benefits

There are no direct benefits for the subjects participating in this study. Known side effects of eplerenone are nausea, diarrhoea, constipation, hypotension, myalgia, myocardial infarction and hyperkalemia. In the EPHESUS trial, the risk of severe hyperkalemia was significantly higher in the eplerenone group. This risk was significantly increased in patients who had loss of kidney function (creatinine clearance <50 mL/min) at baseline. No deaths from hyperkalemia were seen in the eplerenone group. Mean blood pressure increased in both treatment groups, but the increase was limited in eplerenone treated patients. (15) To reduce the risk of hyperkalemia, we exclude patients with a serum potassium of ≥ 4.8 mmol/L (and ≥ 5.1 during treatment) and with renal insufficiency.
In contrast to spironolactone, eplerenone does not affect androgen and progestron receptors. Indeed, in the EPHESUS-study, in which over 6600 patients were included, there was no evidence of an increased incidence of gynecomastia, breast pain or impotence in males.

6.5 Description and justification of route of administration and dosage

Eplerenone is administered orally, in a dose of 50 mg twice daily. A dose of maximal 50 mg daily is registered in patients with clinical evidence of heart failure, after a myocardial infarction, or chronic heart failure. However, several studies investigated the effect of of a total daily dose of ≥100 mg of eplerenone:

In healthy male volunteers, eplerenone has been administered in dosages up to 1000 mg daily during 11 days, compared to a placebo, to examine plasma concentrations and the effect of eplerenone on electrolyte levels. Also, the pharmacodynamic response of 100 mg oral dose of eplerenone once daily for 10 days, was investigated in this study. Treatment with eplerenone 100 mg daily was safe and well-tolerated. There were no significant differences in elektrolyte levels and blood pressure, with respect to baseline levels. (16) Also in patient studies, eplerenone 50 mg bid or dosages exceeding 100 mg daily have been administered before. In patients with a moderate to severe aortic stenosis, patients were allocated to eplerenone 100 mg daily or a placebo to evaluate the effect of eplerenone on the onset of left ventricular dysfunction and left ventricular hypertrophy. Eplerenone did not slow the onset of left ventricular systolic or diastolic dysfunction and did not decrease left ventricle mass. Of 30 patients treated with eplerenone 100 mg daily, 1 patient had to reduce the dosage to 50 mg daily, because of a serum potassium > 5.5 mmol/L and 1 patient withdrawed because of gynecomastia. (17) Another study compared the effect of eplerenone 50 mg daily with eplerenone 100 mg daily, and a placebo on urinary albumin-creatinine ratio (UACR) and blood pressure in patients with type 2 diabetes mellitus. Patients were already treated with enalapril 20 mg once daily and those patients with an UACR of or > 50 mg/g were assigned to one of the 3 treatment groups. No difference in severe hyperkalemia was seen in the treatment groups. Both dosages of eplerenone decreased UACR. Similar decreases of systolic blood pressure were seen in the 3 treatment groups. (18) In patients with hypertension, eplerenone 50-200 mg daily was compared to amlodipine 2.5-10 mg daily. Dosages were raised untill target blood pressure was achieved (SBP <140 mm Hg). Sixty-five % of the patients allocated to eplerenone, were treated with 200mg daily and 15% was administered 100 mg daily. After 24 weeks of treatment, reductions in SBP was similar in the treatment groups. Reduction in DBP was modestly larger in the amlodipine treated group. Sixty-four % of the patients in the eplerenone group reported adverse events, compared to 70% in the amlodipine group. Headache was reported most often (16.4%). Serum potassium >5.5 mmol/L occured in 3% of the eplerenone group, compared to 1.5% in the amlodipine group. Gynecomastia or other hormonal disturbances were not reported, and there were no deaths. (19) The study by Weinberger et al. examined the efficacy, safety and tolerability of eplerenone in patients with mild to moderate hypertension. Patients were randomly assigned to eplerenone 50, 100, 200 or 400 mg once daily; or 25, 50 or 200 mg twice daily, compared to spironolactone 50 mg twice

daily; or placebo. The antihypertensive effect of eplerenone increased in a dose-response manner, with eplerenone 200 mg once daily being optimal. No significant differences in blood pressure were seen between the once and twice daily regimens. The incidence of adverse effects in eplerenone treated patients was similar to the placebo group. One serious drug-related adverse event was reported in the study. This event was seen a patient treated with eplerenone 50 mg once daily. Mean serum potassium increased significantly in patients in the eplerenone 400 mg daily group, in the eplerenone 25, 50 and 100 mg twice daily group, and in the spironolactone group, compared to patients in the placebo group. Seventeen patients had a serum potassium >5.5 mEq/L. This hyperkalemia was reported as an adverse event in only 3 patients (1 in placebo group, 1 in daily 100 mg eplerenone group, and 1 in daily 400 mg eplerenone group), and only 2 patients in the entire study were judged to have clinical significant changes of serum potassium. The authors do not go into detail about why 17 out of 400 patients suffered from hyperkalemia. Blood pressure influencing drugs, as well as loss of renal function (serum creatinine >1.5 mg/dL) and a serum potassium >5.0 mEq/L were included in the exclusion criteria. None of the 17 patients with a serum potassium >5.5 mEq/L withdrew from the study. Gynecomastia or impotence was not reported in the eplerenone groups, but intermenstrual bleeding occured in 1 patient in the spironolactone group. In the eplerenone 400 mg once daily group, a significant increase in TSH level was observed. (20) High dose of eplerenone (100-300 mg daily) has been compared to high dose of spironolactone (75-225 mg daily) in patients with primary hyperaldosteronism. Decrease in both systolic and diastolic blood pressure was lower in the eplerenone treated group. Potassium rose in both groups, although levels did not exceed 4 mmol/L and rise of potassium was significantly greater in the spironolactone group. Twenty-one % of the patients developed gynecomastia in the spironolactone group, compared to 4.5% in the eplerenone group. (21)

Even in children with hypertension, the efficacy and safety of eplerenone has been examined. A total of 304 children, ages 4-16 years, were randomly assigned to eplerenone 25 mg daily, 25 mg twice daily or 50 mg twice daily (phase A) and then allocated to active therapy or a placebo for 4 weeks (phase B). In phase A no dose-relationship regarding blood pressure was seen. Reduction of SBP in only the eplerenone 50 mg twice daily group was significant. Nine subjects had elevated serum potassium levels (5.1 to 8.0 mEq/L), but most values appeared to be normal after a second measurement. The authors do not describe to which groups these 9 children were

allocated. Eplerenone was well tolerated, with comparable percentages of adverse events in the eplerenone and placebo group. (22)
In our study, being a proof-of-principle study, we aim to use a high dose of eplerenone, which has proven to be safe. Given the results of the above described studies, we consider treatment with eplerenone 50 mg bid to be safe in healthy volunteers. Although effective in lowering blood pressure in patients with hypertension (19, 20, 23), eplerenone does not seem to affect blood pressure in healthy volunteers. (16) Importantly, dosages needed to reduce blood pressure were considerably higher than the 50 mg twice daily we will administer. (19, 20) For these reasons, we expect no (signifiant) change in blood pressure in our healthy male volunteers. To confirm this hypothesis, we will monitor blood pressure at baseline and at the end of treatment period (day 7). Duration of treatment will be only 8 days, and serum potassium will be measured before inclusion in the study and twice during treatment (on day 3, 4 or 5, and on day 7 (during the experiment)). (see paragraph 4.3 for exclusion criteria)

We have chosen a treatment duration of 8 days to mimick the design of a previous study of our group in which we demonstrate that rosuvastatine upregulates CD73 activity. (11) We have chosen to administer eplerenone twice daily because the Endocrine Society recommends twice daily administration based on the pharmacokinetic profile. (14)

6.6 Dosages, dosage modifications and method of administration

Please see previous section

6.7 Preparation and labelling of Investigational Medicinal Product

Eplerenone tablets will be over-encapsulated by the department of Clinical Pharmacy of the Radboud University Nijmegen Medical Centre, and further distributed to the Clinical Research Centre Nijmegen, where the drugs will be stocked under GMP-conditions. Fully mimicking placebo will be prepared by the department of Clinical Pharmacy of the Radboud University Nijmegen Medical Centre.

6.8 Drug accountability

The department of Clinical Pharmacy of the Radboud University Medical Centre will provide the eplerenone and placebo used in this trial. The products are transported to the Clinical Research Centre Nijmegen at the RUNMC and stored there under GMP conditions.

7. Non-investigational product

7.1 Name and description of non-investigational product(s)

During the experiments, we will administer several agents into the brachial artery:

1. adenosine
2. dipyridamole
3. caffeine
4. sodium-nitroprusside

All these compounds are given into the brachial artery to ensure a high local concentration in the forearm vascular bed, whereas the systemic circulating concentration is very low to prevent any systemic effects. All these compounds have been used, in the same dosages, in many previous studies from our department. (10-13)

7.2 Summary of findings from non-clinical studies

We kindly refer to the SPC's of the 4 drugs, which are attached (C1.2-C1.6).

7.3 Summary of findings from clinical studies

For details, see SPC text. Adenosine and dipyridamole are currently registered for myocardial stress testing. The dose we use in our study, however, is much lower than the dosages used for these purposes.

7.4 Summary of known and potential risks and benefits

We will administer all drugs into the brachial artery. This approach guarantees an effective concentration into the forearm vascular bed, which induces rapid forearm skeletal muscle vasodilation. In the past decades, we have given these drugs in these dosages very often

into the brachial artery of healthy subjects, and we have never seen any side-effects. Therefore, we are convinced that the use of these compounds is without any risks.

7.5 Description and justification of route of administration and dosage

The following dosages are based on previous studies, performed in our department:

-Dipyridamole 10, 30 and 100 ug/min/dL per forearm with 5 min per dose and 50 uL/min/dL. These dosages we used in a previous study. (10)
-Caffeine 90 ug/min/dL per 100 mL of forearm volume.
-adenosine 1.5 and 5.0 ug/min/dL
-SNP 60 and 600 ng/min/dL

7.6 Dosages, dosage modifications and method of administration

Please see above

7.7 Preparation and labelling of Non Investigational Medicinal Product

All products will be provided by the department of Clinical Pharmacy of the Radboud University Nijmegen Medical Centre and stocked under GMP conditions at the Clinical Research Centre Nijmegen. The final solutions will be prepared by the investigator on the day of the experiment using saline.

7.8 Drug accountability

Please see above

8. Methods

8.1 Study parameters/endpoints

8.1.1 Main study parameter/endpoint

Forearm blood flow response to the intrabrachial administration of incremental dosages of dipyridamole, after treatment with eplerenone, compared to placebo.

8.1.2 Secondary study parameters/endpoints (if applicable)

-Forearm blood flow response to the intrabrachial administration of incremental dosages of dipyridamole, with and without caffeine, after eplerenone treatment.
-Forearm blood flow to incremental periods of arterial occlusion.

8.1.3 Other study parameters (if applicable)

Not applicable.

8.2 Randomisation, blinding and treatment allocation

The study is a double-blinded randomized placebo-controlled cross-over study. Randomisation will be performed by the department of Clinical Pharmacy of the Radboud University Nijmegen Medical Centre. The randomisation code will be kept by this department of Clinical Pharmacy.

8.3 Study procedures

Please see chapter 3 (Study design). In brief, volunteers will be randomly allocated to receive an 8-day treatment with eplerenone and after 4 weeks a fully mimicking placebo or vice versa, in a double-blinded design.
During treatment (day 7), we will measure the vasodilator response to 3 increasing concentrations of intra-arterial dipyridamole, in the absence and presence of intra-arterial infusion of caffeine. PORH to 2 different periods of arterial occlusion will be measured.

During a separate visit (day 8) vasodilator responses to intra-arterially infused sodium nitroprusside and adenosine will be measured.

8.4 Withdrawal of individual subjects

Subjects can leave the study at any time for any reason if they wish to do so without any consequences. The investigator can decide to withdraw a subject from the study for urgent medical reasons.

8.4.1 Specific criteria for withdrawal (if applicable)

Subjects are excluded from the trial when the serum potassium concentration exceed 5.0 mmol/l (≥ 5.1 mmol/L) on day 3, 4, or 5 of treatment, as mentioned previously.

8.5 Replacement of individual subjects after withdrawal

In this study, we will include 14 subjects: two subjects more than the number of subjects needed according to our power analyses to account for drop-outs.

8.6 Follow-up of subjects withdrawn from treatment

A participant who decides to withdraw from the trial will be invited to an exit-interview concerning their withdrawal. In subjects excluded because of an increased serum potassium concentration we will draw blood after each two days, to monitor the potassium concentration until this is <5.0 mmol/L.

8.7 Premature termination of the study

Not applicable

9. Safety reporting

9.1 Section 10 WMO event

In accordance to section 10, subsection 1, of the WMO, the investigator will inform the subjects and the reviewing accredited METC if anything occurs, on the basis of which it appears that the disadvantages of participation may be significantly greater than was foreseen in the research proposal. The study will be suspended pending further review by the accredited METC, except insofar as suspension would jeopardise the subjects' health. The investigator will take care that all subjects are kept informed.

9.2 AEs, SAEs and SUSARs

9.2.1 Adverse events (AEs)

Adverse events are defined as any undesirable experience occurring to a subject during the study, whether or not considered related to the investigational product / the experimental intervention. All adverse events reported spontaneously by the subject or observed by the investigator or his staff will be recorded.

9.2.2 Serious adverse events (SAEs)

A serious adverse event is any untoward medical occurrence or effect that at any dose:

- results in death;
- is life threatening (at the time of the event);
- requires hospitalisation or prolongation of existing inpatients' hospitalisation;
- results in persistent or significant disability or incapacity;
- is a congenital anomaly or birth defect;
- Any other important medical event that may not result in death, be life threatening, or require hospitalization, may be considered a serious adverse

experience when, based upon appropriate medical judgement, the event may jeopardize the subject or may require an intervention to prevent one of the outcomes listed above.

All serious adverse events will be reported to the sponsor of this trial: Prof. F. Russel, head of the department of Pharmacology and Toxicology. He delegates the appropriate handling of these events to the coordinating and principle investigators of this trial: Dr. G. A. Rongen and Dr. N.P. Riksen, Further report of these adverse events will be performed according to GCP as outlined below.

The sponsor will report the SAEs through the web portal *ToetsingOnline* to the accredited METC that approved the protocol, within 15 days after the sponsor has first knowledge of the serious adverse reactions.

SAEs that result in death or are life threatening should be reported expedited. The expedited reporting will occur not later than 7 days after the responsible investigator has first knowledge of the adverse reaction. This is for a preliminary report with another 8 days for completion of the report.

9.2.3 Suspected unexpected serious adverse reactions (SUSARs)

Adverse reactions are all untoward and unintended responses to an investigational product related to any dose administered.

Unexpected adverse reactions are SUSARs if the following three conditions are met:

1. the event must be serious (see chapter 9.2.2);
2. there must be a certain degree of probability that the event is a harmful and an undesirable reaction to the medicinal product under investigation, regardless of the administered dose;
3. the adverse reaction must be unexpected, that is to say, the nature and severity of the adverse reaction are not in agreement with the product information as recorded in:
   - Summary of Product Characteristics (SPC) for an authorised medicinal product;

- Investigator's Brochure for an unauthorised medicinal product.

The sponsor will report expedited the following SUSARs through the web portal *ToetsingOnline* to the METC:

- SUSARs that have arisen in the clinical trial that was assessed by the METC;
- SUSARs that have arisen in other clinical trials of the same sponsor and with the same medicinal product, and that could have consequences for the safety of the subjects involved in the clinical trial that was assessed by the METC.

The remaining SUSARs are recorded in an overview list (line-listing) that will be submitted once every half year to the METC. This line-listing provides an overview of all SUSARs from the study medicine, accompanied by a brief report highlighting the main points of concern.

The expedited reporting of SUSARs through the web portal ToetsingOnline is sufficient as notification to the competent authority.

The sponsor will report expedited all SUSARs to the competent authorities in other Member States, according to the requirements of the Member States.

The expedited reporting will occur not later than 15 days after the sponsor has first knowledge of the adverse reactions. For fatal or life threatening cases the term will be maximal 7 days for a preliminary report with another 8 days for completion of the report.

If a SAE or SUSAR occurs and there is the need to know whether a patient was treated with a mineralocorticoid receptor antagonist or placebo, the pharmacist on call can be contacted (24 hours a day, 7 days a week) and he/she will be able to break the blinding code for that particular participant.

9.3 Annual safety report

In addition to the expedited reporting of SUSARs, the sponsor will submit, once a year throughout the clinical trial, a safety report to the accredited METC, competent authority, and competent authorities of the concerned Member States.

This safety report consists of:

- a list of all suspected (unexpected or expected) serious adverse reactions, along with an aggregated summary table of all reported serious adverse reactions, ordered by organ system, per study;
- report concerning the safety of the subjects, consisting of a complete safety analysis and an evaluation of the balance between the efficacy and the harmfulness of the medicine under investigation.

9.4 Follow-up of adverse events

All AEs will be followed until they have abated, or until a stable situation has been reached. Depending on the event, follow up may require additional tests or medical procedures as indicated, and/or referral to the general physician or a medical specialist.

SAEs need to be reported till end of study within the Netherlands, as defined in the protocol

9.5 [Data Safety Monitoring Board (DSMB) / Safety Committee]

Not applicable

10. Statistical analysis

10.1 Primary study parameter(s)

The FBF response to the administration of dipyridamole after treatment with eplerenone compared to placebo will be log transformed and evaluated using a linear mixed-model analysis with random factor subject, fixed factors dipyramole dose, eplerenone (versus placebo), dipyramole dose * eplerenone, and period. The logarithm of the baseline FBFs will be included as co-variables. The results will be back-transformed and 95% confidence intervals will be presented. A two-sided p-value of < 0.05 will be considered to be statistically significant.

10.2 Secondary study parameter(s)

The reduction in FBF response, due to the administration of caffeine in the presence of dipyridamole and eplerenole, will be analysed in a similar way, using a linear mixed-model analysis with random factor subject, fixed factors dipyramole dose, caffeine (yes/no), dipyramole dose * caffeine, and period. The logartithm of the baseline FBF will be included as co-variables. The dependent variable will be the log-transformed FBF after eplenerone administration (with and without caffeine). Other endpoints will be analysed using similar random effects models. A p-value of < 0.05 will be considered to be statistically significant.

10.3 Other study parameters

Not applicable

10.4 Interim analysis (if applicable)

Not applicable

11. Ethical considerations

11.1 Regulation statement

This study will be conducted according to the principles of the Declaration of Helsinki (version: April 2008) and in accordance with the Medical Research Involving Human Subjects Act (WMO) and other guidelines and regulations.

11.2 Recruitment and consent

Healthy volunteers will be recruited at the University Medical Centre Nijmegen (please see chapter 4). Informed consent will be achieved by a ‘participation information letter’ and potential participants will be given the opportunity to address their questions to the investigator. Participation is only possible after written informed consent. Please find the patient information letter and informed consent form as separate documents.

11.3 Objection by minors or incapacitated subjects (if applicable)

Not applicable

11.4 Benefits and risks assessment, group relatedness

The study does not concern minors or incapacitated subjects. The subjects participating in this study do not benefit from participation. The risks of participation are low (please see paragraph 6.4). The results of this study increase knowledge about the mechanism of action of MR antagonists on blood flow response. This can potentially be used in the future to guide treatment of patients with an increased risk for (myocardial) ischemia reperfusion. The potential benefits of this study outweighs the small risks of treatment with eplerenone for our subjects.

11.5 Compensation for injury

The sponsor/investigator has a liability insurance which is in accordance with article 7, subsection 6 of the WMO.

11.6 Incentives (if applicable)

The subjects will receive 300 euros as a compensation for participation in the study. Should a subject withdraw after the first experiment, 150 euros will be paid. If the investigator decides to withdraw a subject from the study, because of hyperkalemia or side effects of eplerenone that cannot be controlled by simple interventions (such as paracetamole), the full amount of 300 euros will be paid.

12. Administrative aspects, monitoring and publication

12.1 Handling and storage of data and documents

All research outcomes will be archived in the personal medical file of each participant. This medical file will not leave the Radboud University Nijmegen Medical Centre. This source document can only be viewed by trial monitors and investigators involved. The following data will be archived in the source document: copy of signed informed consent form, EKG, subject study code, each visit date and all study outcomes (laboratory) and possible adverse events. In addition to the source document, a case report form (CRF) will be completed. The CRF is anonymized (only contains the subject study code) and may leave the hospital for data management or monitoring purposes. A copy of this CRF remains at the RUNMC for 15 years.

The following data will be archived in the CRF: subject code; date, all study outcomes (laboratory) and possible adverse events, (serious) adverse events. A subject code log will be archived in the trial master file. Furthermore we will file the drug accountability forms in the trial master file together with the approved version of the protocol, correspondence with the METC and CVs of all involved investigators.

12.2 Monitoring and Quality Assurance

According to the NFU publication "kwaliteitsborging van mensgebonden onderzoek", the participants of our study have a small chance of moderate risk. However, we believe that given the complexity of the experiment, a moderate intensive monitoring according to the NFU guideline, is warranted. Monitoring will be performed by a BROK-certified researcher of the UMCN, which will be coordinated by the CRCN.

According to the NFU guidelines, three visits will be performed (initiation, one after inclusion of half of the participants, and one closure visit). Informed consent, SAE's and SUSAR's will be checked for all participants. In-, and exclusion criteria will be checked for the first 7 participants. A complete source data verification will be performed for 4 subjects. Please find attached at the end of the protocol a separate monitoring plan. (C1.7)

12.3 Amendments

A ‘substantial amendment’ is defined as an amendment to the terms of the METC application, or to the protocol or any other supporting documentation, that is likely to affect to a significant degree:

- the safety or physical or mental integrity of the subjects of the trial;
- the scientific value of the trial;
- the conduct or management of the trial; or
- the quality or safety of any intervention used in the trial.

All substantial amendments will be notified to the METC and to the competent authority.

Non-substantial amendments will not be notified to the accredited METC and the competent authority, but will be recorded and filed by the sponsor.

12.4 Annual progress report

The sponsor/investigator will submit a summary of the progress of the trial to the accredited METC once a year. Information will be provided on the date of inclusion of the first subject, numbers of subjects included and numbers of subjects that have completed the trial, serious adverse events/ serious adverse reactions, other problems, and amendments.

12.5 End of study report

The sponsor will notify the accredited METC and the competent authority of the end of the study within a period of 90 days. The end of the study is defined as the last patient’s last visit.

In case the study is ended prematurely, the sponsor will notify the accredited METC and the competent authority within 15 days, including the reasons for the premature termination.

Within one year after the end of the study, the investigator/sponsor will submit a final study report with the results of the study, including any publications/abstracts of the study, to the accredited METC and the Competent Authority.

12.6 Public disclosure and publication policy

Before initiation of the study, the study details will be published on ClinicalTrials.gov. After completion of the study, the results will be submitted to a scientific journal for publication.

13. Structured risk analysis

13.1 Potential issues of concern

a. Level of knowledge about mechanism of action

The mechanism of action of eplerenone in the kidney is known and described in detail in the SPC which is attached. In this study, we aim to investigate whether eplerenone also activates CD73. This hypothesis is based on a recent animal study, which is described in more detail in chapter 1 of this protocol. (2)

b. Previous exposure of human beings with the test product(s) and/or products with a similar biological mechanism

Eplerenone has been widely used and is registered for the treatment of patients with heart failure. In the USA, eplerenone is approved in primary hypertension since 2002. In addition, eplerenone is used in the medical treatment of patients with primary hyperaldosteronism.

c. Can the primary or secondary mechanism be induced in animals and/or in *ex-vivo* human cell material?

The aim of our study is to translate previous in vitro findings and findings in animal studies to the human in vivo situation. Therefore, our study has to be conducted in humans.

d. Selectivity of the mechanism to target tissue in animals and/or human beings

Eplerenone is a selective MR-antagonist. See also SPC.

e. Analysis of potential effect

The primary endpoint of the study is the forearm blood flow response to the administration of dipyridamole into the brachial artery. This is detected by venous occlusion

plethysmography of the forearm. The vasodilator response to dipyridamole reflects adenosine formation by CD73.

f. Pharmacokinetic considerations

We would like to refer to the SPC and to paragraph 6.5 of this protocol for a detailed description of the pharmacokinetic properties of eplerenone.

g. Study population

The study is performed in healthy male volunteers.

h. Interaction with other products

Plasma concentrations of eplerenone can increase and side effects can be potentiated during co-administration of CYP3A4 inhibitors. NSAIDs increase the risk of acute kidney failure. Therefore, no co-medication is allowed in our volunteers.

i. Predictability of effect

Not applicable

j. Can effects be managed?

Not applicable

13.2 Synthesis

Given:

-the fact that we use healthy male volunteers

- administration of eplerenone to healthy male subjects without hyperkalemia (serum potassium < 4.8 mmol/L)

- screening of potassium twice during each treatment (eplerenone and placebo) and discontinuing the study in patients who reached a serum potassium of ≥ 5.1 mmol/L

- monitoring blood pressure

-and a 27 gauge needle used for intrabrachial administration of dipyridamole, caffeine, adenosine and sodium-nitroprusside

we think that the potential risks for the volunteers are low. Also, the methods used (venous blood drawing, venous occlusion plethysmography, and insertion of a cannula into the brachial artery) are well-established and have been performed very often by our research group, without any serious events.

14. References

1. Loan Le TY, Mardini M, Howell VM, Funder JW, Ashton AW, Mihailidou AS. Low-dose spironolactone prevents apoptosis repressor with caspase recruitment domain degradation during myocardial infarction. Hypertension. 2012;59(6):1164-9. Epub 2012/04/18.
2. Schmidt K, Tissier R, Ghaleh B, Drogies T, Felix SB, Krieg T. Cardioprotective effects of mineralocorticoid receptor antagonists at reperfusion. European heart journal. 2010;31(13):1655-62. Epub 2009/12/24.
3. Mihailidou AS, Loan Le TY, Mardini M, Funder JW. Glucocorticoids activate cardiac mineralocorticoid receptors during experimental myocardial infarction. Hypertension. 2009;54(6):1306-12. Epub 2009/10/21.
4. Chai W, Garrelds IM, de Vries R, Danser AH. Cardioprotective effects of eplerenone in the rat heart: interaction with locally synthesized or blood-derived aldosterone? Hypertension. 2006;47(4):665-70. Epub 2006/02/24.
5. van den Borne SW, Isobe S, Zandbergen HR, Li P, Petrov A, Wong ND, et al. Molecular imaging for efficacy of pharmacologic intervention in myocardial remodeling. JACC Cardiovascular imaging. 2009;2(2):187-98. Epub 2009/04/10.
6. Kessler-Icekson G, Schlesinger H, Freimann S, Kessler E. Expression of procollagen C-proteinase enhancer-1 in the remodeling rat heart is stimulated by aldosterone. The international journal of biochemistry & cell biology. 2006;38(3):358-65. Epub 2005/11/23.
7. Ramakers BP, Pickkers P, Deussen A, Rongen GA, van den Broek P, van der Hoeven JG, et al. Measurement of the endogenous adenosine concentration in humans in vivo: methodological considerations. Current drug metabolism. 2008;9(8):679-85. Epub 2008/10/16.
8. Moser GH, Schrader J, Deussen A. Turnover of adenosine in plasma of human and dog blood. The American journal of physiology. 1989;256(4 Pt 1):C799-806. Epub 1989/04/01.
9. Riksen NP, Rongen GA. Targeting adenosine receptors in the development of cardiovascular therapeutics. Expert review of clinical pharmacology. 2012;5(2):199-218. Epub 2012/03/07.
10. Bijlstra P, van Ginneken EE, Huls M, van Dijk R, Smits P, Rongen GA. Glyburide inhibits dipyridamole-induced forearm vasodilation but not adenosine-induced forearm vasodilation. Clinical pharmacology and therapeutics. 2004;75(3):147-56. Epub 2004/03/06.
11. Meijer P, Wouters CW, van den Broek PH, de Rooij M, Scheffer GJ, Smits P, et al. Upregulation of ecto-5'-nucleotidase by rosuvastatin increases the vasodilator response to ischemia. Hypertension. 2010;56(4):722-7. Epub 2010/08/04.
12. Meijer P, Wouters CW, van den Broek PH, Scheffer GJ, Riksen NP, Smits P, et al. Dipyridamole enhances ischaemia-induced reactive hyperaemia by increased adenosine receptor stimulation. British journal of pharmacology. 2008;153(6):1169-76. Epub 2008/02/12.
13. Meijer P, Oyen WJ, Dekker D, van den Broek PH, Wouters CW, Boerman OC, et al. Rosuvastatin increases extracellular adenosine formation in humans in vivo: a new perspective on cardiovascular protection. Arteriosclerosis, thrombosis, and vascular biology. 2009;29(6):963-8. Epub 2009/04/11.
14. Funder JW, Carey RM, Fardella C, Gomez-Sanchez CE, Mantero F, Stowasser M, et al. Case detection, diagnosis, and treatment of patients with primary aldosteronism: an endocrine society clinical practice guideline. The Journal of clinical endocrinology and metabolism. 2008;93(9):3266-81. Epub 2008/06/17.

15. Pitt B, Remme W, Zannad F, Neaton J, Martinez F, Roniker B, et al. Eplerenone, a selective aldosterone blocker, in patients with left ventricular dysfunction after myocardial infarction. The New England journal of medicine. 2003;348(14):1309-21. Epub 2003/04/02.
16. Eudy RJ, Sahasrabudhe V, Sweeney K, Tugnait M, King-Ahmad A, Near K, et al. The use of plasma aldosterone and urinary sodium to potassium ratio as translatable quantitative biomarkers of mineralocorticoid receptor antagonism. Journal of translational medicine. 2011;9:180. Epub 2011/10/25.
17. Stewart RA, Kerr AJ, Cowan BR, Young AA, Occleshaw C, Richards AM, et al. A randomized trial of the aldosterone-receptor antagonist eplerenone in asymptomatic moderate-severe aortic stenosis. American heart journal. 2008;156(2):348-55. Epub 2008/07/29.
18. Epstein M, Williams GH, Weinberger M, Lewin A, Krause S, Mukherjee R, et al. Selective aldosterone blockade with eplerenone reduces albuminuria in patients with type 2 diabetes. Clinical journal of the American Society of Nephrology : CJASN. 2006;1(5):940-51. Epub 2007/08/19.
19. White WB, Duprez D, St Hillaire R, Krause S, Roniker B, Kuse-Hamilton J, et al. Effects of the selective aldosterone blocker eplerenone versus the calcium antagonist amlodipine in systolic hypertension. Hypertension. 2003;41(5):1021-6. Epub 2003/04/19.
20. Weinberger MH, Roniker B, Krause SL, Weiss RJ. Eplerenone, a selective aldosterone blocker, in mild-to-moderate hypertension. American journal of hypertension. 2002;15(8):709-16. Epub 2002/08/06.
21. Parthasarathy HK, Menard J, White WB, Young WF, Jr., Williams GH, Williams B, et al. A double-blind, randomized study comparing the antihypertensive effect of eplerenone and spironolactone in patients with hypertension and evidence of primary aldosteronism. Journal of hypertension. 2011;29(5):980-90. Epub 2011/04/01.
22. Li JS, Flynn JT, Portman R, Davis I, Ogawa M, Shi H, et al. The efficacy and safety of the novel aldosterone antagonist eplerenone in children with hypertension: a randomized, double-blind, dose-response study. The Journal of pediatrics. 2010;157(2):282-7. Epub 2010/04/20.
23. Jansen PM, Frenkel WJ, van den Born BJ, de Bruijne EL, Deinum J, Kerstens MN, et al. Determinants of blood pressure reduction by eplerenone in uncontrolled hypertension. Journal of hypertension. 2012. Epub 2012/12/20.
